# Tunable transport property of oxygen ion in metal oxide thin film: Impact of electrolyte orientation on conductivity

**DOI:** 10.1038/s41598-017-03705-w

**Published:** 2017-06-14

**Authors:** P. Arunkumar, R. Ramaseshan, S. Dash, K. Suresh Babu

**Affiliations:** 1 Centre for Nanoscience and Technology, Madanjeet School of Green Energy Technology, Pondicherry University, Puducherry, 605 014 India; 2 Thin Film and Coating Section, Surface & Nanoscience Division, Indira Gandhi Centre for Atomic Research (IGCAR), Kalpakkam, 603 102 India

## Abstract

Quest for efficient ion conducting electrolyte thin film operating at intermediate temperature (~600 °C) holds promise for the real-world utilization of solid oxide fuel cells. Here, we report the correlation between mixed as well as preferentially oriented samarium doped cerium oxide electrolyte films fabricated by varying the substrate temperatures (100, 300 and 500 °C) over anode/ quartz by electron beam physical vapor deposition. Pole figure analysis of films deposited at 300 °C demonstrated a preferential (111) orientation in out-off plane direction, while a mixed orientation was observed at 100 and 500 °C. As per extended structural zone model, the growth mechanism of film differs with surface mobility of adatom. Preferential orientation resulted in higher ionic conductivity than the films with mixed orientation, demonstrating the role of growth on electrochemical properties. The superior ionic conductivity upon preferential orientation arises from the effective reduction of anisotropic nature and grain boundary density in highly oriented thin films in out-of-plane direction, which facilitates the hopping of oxygen ion at a lower activation energy. This unique feature of growing an oriented electrolyte over the anode material opens a new approach to solving the grain boundary limitation and makes it as a promising solution for efficient power generation.

## Introduction

Solid oxide fuel cells (SOFCs) is an electrochemical energy conversion devices for the generation of energy with high efficiency (~70%) and possess the advantage of fuel flexibility^1^. In order to facilitate the significant ion diffusion across the electrolyte, SOFC needs to be operated at higher temperatures (~1000 °C). However, at higher temperatures rapid degradation of SOFC components occur, thereby limiting the lifetime as well as the potential for commercialization. Thus, reducing the operating temperature becomes a critical impediment to expanding the applicability of SOFC. However, lowering the operating temperature drastically reduces the ionic conductivity of electrolyte and hence the overall efficiency of the cell^1^. Various approaches have been adopted to improve the ionic conductivity that includes the development of other alternate electrolyte material^2–4^. For the past three decades, doped cerium oxides (CeO2) is widely being investigated as an electrolyte for intermediate temperature SOFC (ITSOFC, 500–750 °C) operation due to its lower activation energy (0.84 eV, single crystal) arising from reduced enthalpy of oxygen ion migration than that of conventional yttria stabilized zirconia (YSZ, ~1.04 eV, single crystal)^5–9^. For example, at ~400 °C, the ionic conductivity of YSZ (8 mol%) has been reported to be 10^−4^ S cm^−1^, while samarium doped cerium (SDC, 20 mol%) oxide exhibits a higher conductivity of 10^−3^ S cm^−1^ ^10^. Doped cerium oxide has cubic fluorite structure which is stable from room temperature to the melting point^11^. Cubic fluorite-type oxides have the general formula of AX_2_, where the A and X-sites are occupied by Ce and O atoms, respectively. The partial substitution of the A-site cation with a trivalent dopant (M^3+^) results in the creation of oxygen vacancy (${V}_{o}^{\cdot \cdot }$) as represented by Krӧger-Vink notation

1 $${M}_{2}{O}_{3}\,\mathop{\longrightarrow }\limits^{Ce{O}_{2}}\,2M^{\prime} ce+3{O}_{0}^{x}+{V}_{O}^{\cdot \cdot }$$

where, ${O}_{O}^{x}$ denotes O ion occupies the oxygen lattice with neutral charge, $2{M}_{Ce}^{^{\prime} }$ denotes M ion occupies the Ce lattice with negative single charge and ${V}_{o}^{\cdot \cdot }$ denotes a oxygen vacancy with double positive charge. The increase in oxygen vacancy concentration upon doping in cerium oxide lattice facilitates the diffusion of oxygen ion by lowering the migration enthalpy, thereby improving the ionic conductivity required for ITSOFC operation^12^.

Though several researchers have demonstrated the potential of doped cerium oxide electrolyte in bulk form for ITSOFC, the thickness of electrolyte in few hundreds of micrometers generates significant ohmic resistance, thereby lowering the overall ionic conduction^13, 14^. Diminution of electrolyte thickness to the range between few micrometers (<~4 μm) to nanometric level in the form of thin film lowers the ohmic loss and augments the ionic conduction due to the shortened distance for ion migration^15–17^. In addition to reduction in electrolyte thickness, engineering interface property of the thin film is equally important in improving the ionic conductivity^18^. Grain boundaries are one of the major interfacial properties of the films which limit the movement of oxygen ion through the electrolyte at an intermediate temperature. In general, activation energy for the grain boundary (~0.90 to 1.61 eV) conduction is higher than that of grain (~0.47 to 0.74 eV) in doped ceria system^19, 20^. The sluggish mobility of ions across the boundaries arises from the presence of space charge layer and impurity/dopant segregation in its vicinity^21, 22^. Thus, considerable effort has been made to reduce the grain boundary area in the electrolyte by growing epitaxial or preferentially oriented film towards a particular direction in order to improve the ionic conductivity^23^. For example, (100) orientated samarium (20 mol%) doped cerium oxide (SDC) film deposited on (001) MgO single crystal exhibited a conductivity value of 0.07 Scm^−1^ at 700 °C which is higher than that of polycrystalline SDC (0.02 Scm^−1^ at 700 °C)^24^. This enhancement in conductivity of textured or epitaxial films resulting from the reduction or absence of grain boundaries, which means the blocking effect of oxygen ion migration near to the grain boundary is relatively reduced as compared to the polycrystalline material (bulk). Therefore, engineering the orientation of the films assume importance in order to enhance the ionic conductivity. So far, the majority of the work reported on tailoring the orientation of electrolyte film has been carried out using single crystal substrate^25, 26^. However, to realize the potential of SOFC towards practical application, the oriented film should be either grown over an anode or a cathode substrate. Theoretical^27^ and experimental^28^ studies have been carried out on growing the electrolyte films over a polycrystalline or amorphous substrate. It has been demonstrated that optimizing the deposition parameters in physical vapor deposition (PVD) technique may result in the textured film on a polycrystalline or amorphous substrate^22^. Electron beam physical vapor deposition (EB-PVD) technique is one of the promising methods in which the thin film parameters such as density, the thickness and orientation can be precisely controlled by tuning the deposition parameters. In our previous work, we demonstrated the effect of a change in target material property in tuning the orientation of samarium doped cerium oxide film on an amorphous substrate in EB-PVD technique^29^.

Here, we report the fabrication of textured SDC film over polycrystalline (anode) and amorphous (quartz) substrates by the varying the substrate temperature through EB-PVD technique. The influence of substrate temperature on the orientation of film has been discussed in detail using extended structural zonal model (ESZM) proposed by Mahieu *et al*.^27^. The in-plane conductivity measurement reveals a strong influence of orientation on ionic conductivity. To the best of our knowledge, this is the first report which explains the possible mechanism for preferential orientation over the anode substrate and its potential impact on ionic transport for ITSOFC application.

## Results and Discussion

### Structural analysis

**Figure 1:**
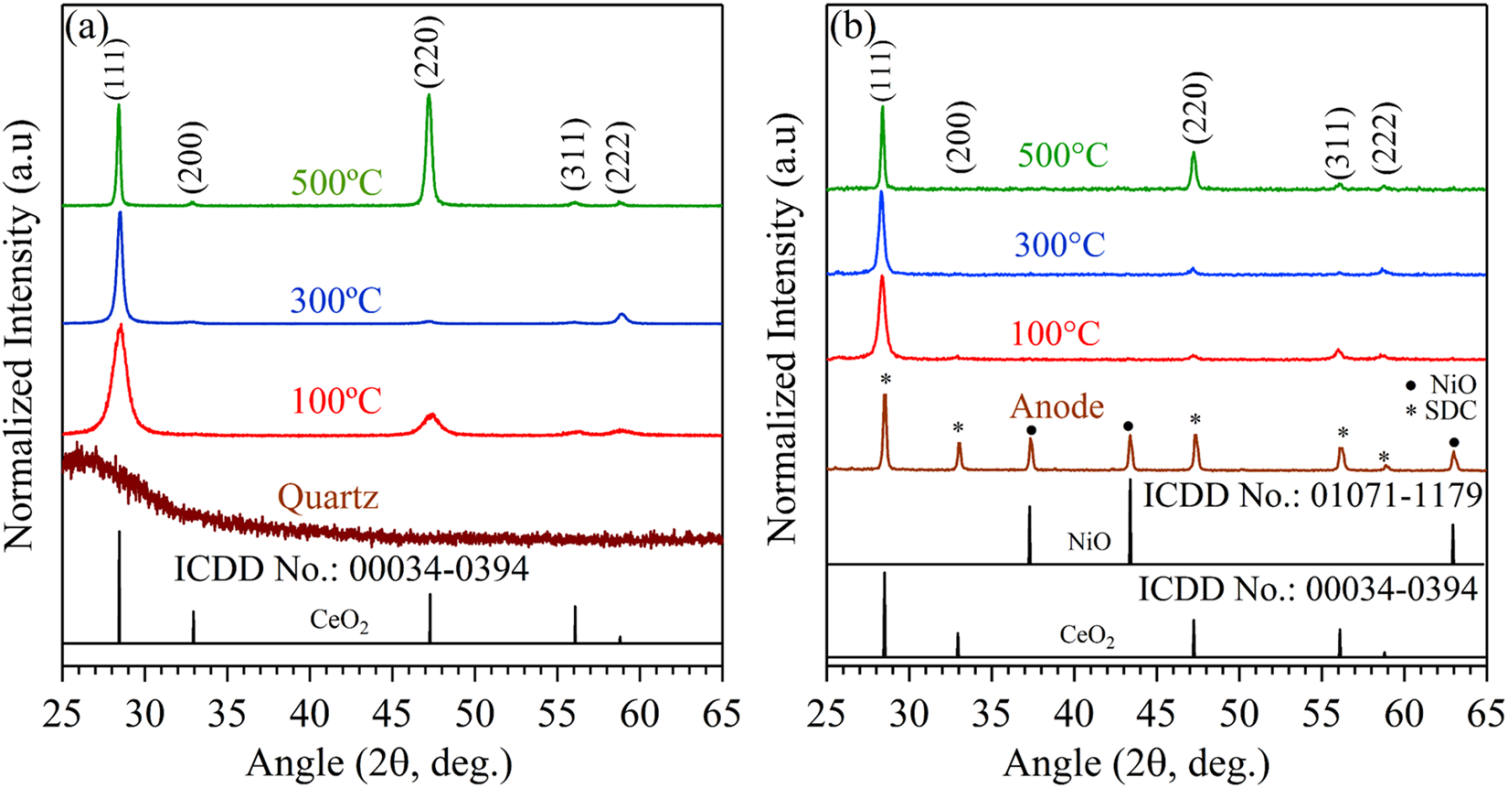
B-B geometry XRD pattern of the thin film deposited at substrate temperatures of 100, 300 and 500 °C over Quartz (**a**) and Anode (**b**).

Thin films are two or three-dimensional arrangement of atoms over the substrate surface. Symmetric reflection X-ray scan (B-B geometry, *θ*-*2θ*) provides the crystallographic information in out-of-plane direction. XRD pattern of the films deposited on quartz and anode substrate at a different deposition temperature are shown in Fig. 1(a) and (b). Independent of substrate nature and deposition temperature, the XRD pattern confirms the presence of the single phase cubic fluorite structure of CeO_2_ (ICDD No.: 00034-0394) without any additional impurity phase. Interestingly, the relative intensities of reflection planes varied in response to the substrate nature and the deposition temperature.

**Figure 2:**
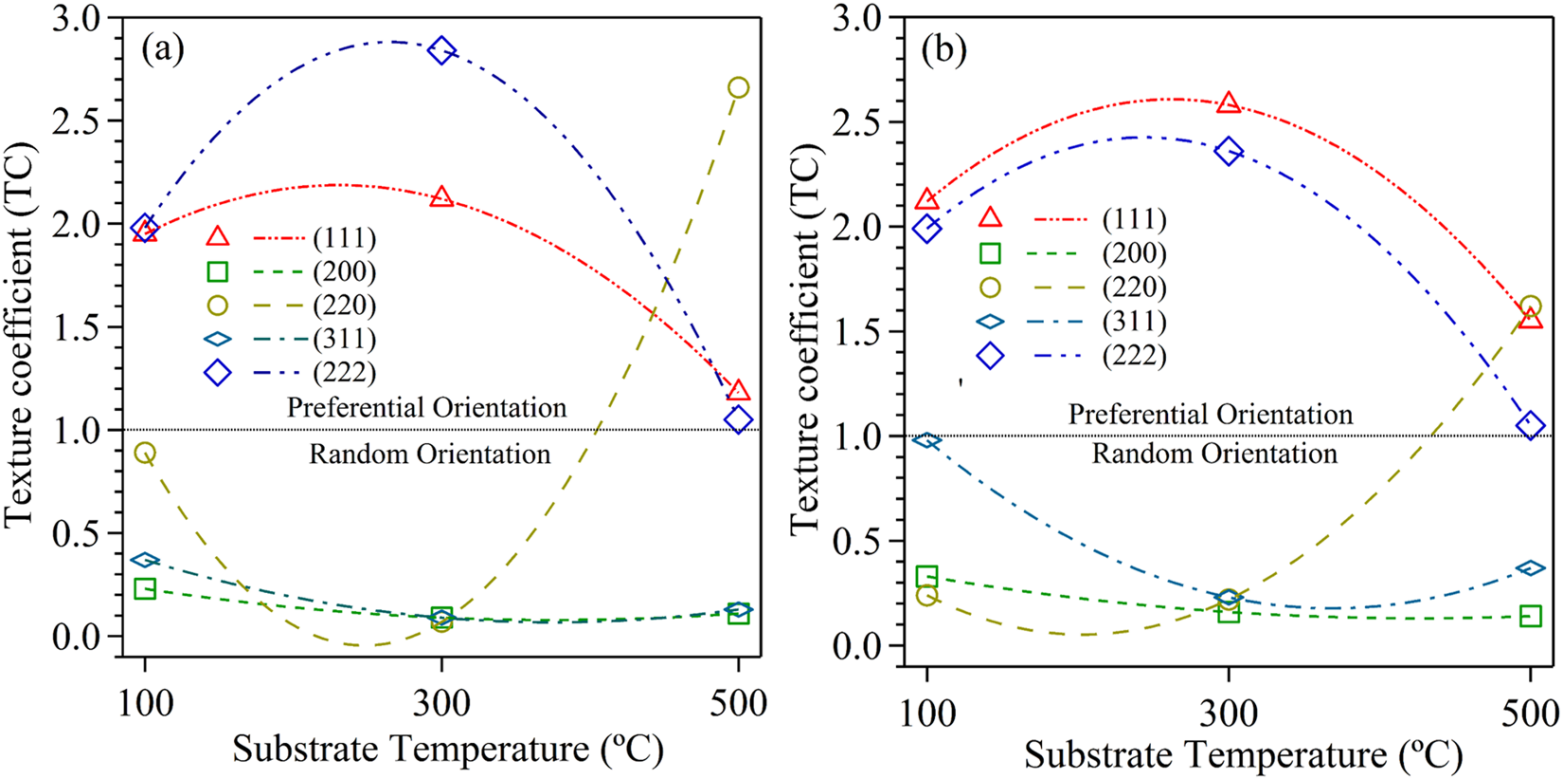
Texture Coefficient of the thin film with respect to the thin film deposited at a different substrate temperature (**a**) Quartz and (**b**) Anode.

XRD pattern of the films deposited at 100 and 300 °C showed an intense peak of (111) plane in comparison to standard ICDD pattern indicates the presence of preferential orientation in (111) direction. In addition to the intense (111) plane, weak and low intense (220), (311) and (222) plane was observed at 100 °C. On the other hand, only (111) and (222) peaks were present prominently at 300 °C. For the films deposited at 500 °C, an intense (220) plane along with (111) plane indicates the presence of mixed (111) and (220) orientation in out-of-plane direction. Thus, the X-ray results confirm the strong preferential orientation in (111) direction for the film deposited at 300 °C as compared to the films deposited at 100 and 500 °C. Harris equation was used to evaluate the degree of preferential orientation in terms of texture coefficient (TC)^23^.

2 $${T}_{hkl}(TC)=\,\frac{{I}_{hkl}\,/{I}_{hkl}^{\ast }}{\frac{1}{n}\sum [{I}_{hkl}/{I}_{hkl}^{\ast }]}$$

where, *I*_*hkl*_ denotes the experimental intensity obtained from the XRD peak of a particular orientation, *I* ^*^_hkl_ denotes the ICDD intensity of the corresponding plane, and *n* was the number of peaks used for calculation. The diffracted peaks of (111), (200), (220), (311) and (222) were fitted with Voigt function to find the precise integrated intensity to calculate the TC value. Figure 2(a) and (b) shows the TC value for the films deposited on quartz and anode substrate, respectively, with respect to the substrate temperature. According to Harris equation, an increase in TC value beyond one (represented by black colored dashed line in Fig. 2) for a particular plane specifies the preferential growth along that particular direction by dominating all other crystallographic orientation in comparison to bulk (based on ICDD reference card). Thus, higher TC value (>1) for a particular plane refers to the greater degree of preferential orientation along that specific plane in the out-of-plane direction. TC value for (111) and (222) planes was found to be more than one with increasing trend of substrate temperature till 300 °C and subsequently decreased at 500 °C on both the substrates. The plane (220) exhibited an opposite behavior to that of (111), *i*.*e*., a decreasing trend in TC value upto 300 °C, but a higher TC value was observed at 500 °C. In particular, the films deposited at 300 °C exhibits the highest TC value for both (111) and (222) planes specifies the alignment of a larger fraction of grains towards the (111) direction. At 500 °C, the TC value for (220) plane increased along with (111) and (222) planes denotes to the grain growth favored in the both the direction leading to the mixed orientation. Thus, the quantitative analysis of TC value shows the film deposited at 300 °C exhibits a strong preferential orientation in (111) direction in comparison to the films deposited at 100 and 500 °C.

**Figure 3:**
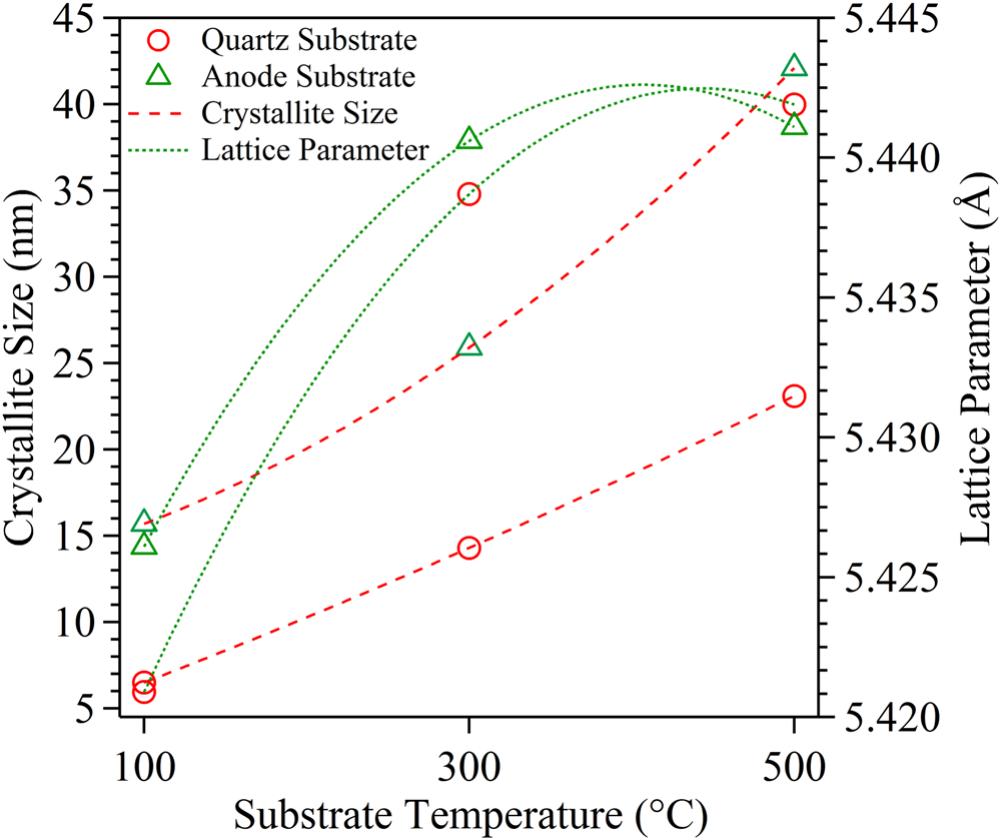
Calculated crystallite size using Scherrer equation for thin film with respect to substrate temperature deposited on quartz and anode.

The mean crystallite size, calculated using Scherrer equation and the change in lattice parameter as a function of substrate temperature are shown in Fig. 3. The crystallite size increases with increase in deposition temperature on both the substrate. Relatively, the change in crystallite size was found to be higher for the film deposited over anode substrate (16 to 43 nm) as compared to quartz substrate (7 to 23 nm). Since, all the processing parameters were kept constant, except that of substrate temperature. The observed increase in size with temperature can be presumed to arise from the enhancement in diffusion co-efficient (*D*) of the evaporated atom over the substrate surface as given by equation 4^30^.

3 $${\rm{D}}={D}_{o}\,{e}^{\frac{-{\rm{\Delta }}E}{kT}}$$

where *D*_*o*_ represent the macroscopic diffusivity of adatom and assumed to be in the order of 10^−3^ cm^2^/s, as proposed by Tsong, T.T.^31^, *ΔE* is activation energy for diffusion, *k is* Boltzmann constant, and *T* is absolute temperature of the substrate. Increase (decrease) in substrate temperature, in turn, increases (decreases) the diffusion co-efficient of the incoming adatom on the substrate. Silly *et al*., evaluated the *ΔE* value for Ce adatom as 11 meV using scanning tunneling microscopy^32^. From equation (3) the calculated diffusion coefficients (D) for the films deposited at 100, 300 and 500 °C was found to be 7.1 × 10^−3^, 8 × 10^−3^, and 8.4 × 10^−3^ cm^2^/s, respectively. Thus, an increase in D with the substrate temperature, in turn, increases the average displacement of adatom from initial to final site on the 2D surface^33, 34^. Accordingly, larger displacement of adatom increases the crystallite size of the film deposited at higher substrate temperature for both the substrates (Fig. 3). Among the substrates, the film deposited on anode substrate exhibited a higher crystallite size than the quartz indicates the contribution of substrate property over the average displacement of adatom during the thin film formation. The anode is a composite material consisting of NiO and SDC in the weight ratio of 40:60. The presence of SDC in anode increases the lateral interaction between the adatom and substrate during the growth process of the thin film. This miscibility of Ce and Sm atom with the anode substrate intensifies the nucleation process of adatom, which in turn increase the size of the 2D island formation over the anode substrate than that of quartz^35^. Thus, the increase in island size over the anode substrate lead to a higher crystallite size as compared to quartz.

Lattice parameter (a) was calculated using Nelson-Riley function (equation 4) in order to minimize the strain effect^36^.

4 $$F(\theta )=\frac{1}{2}\,(\frac{{\cos }^{2}\theta }{\sin \,\theta }+\,\frac{{\cos }^{2}\theta }{\theta })$$

where, *θ* is the Bragg angle. A graph was made by plotting *a* from all the (*hkl*) reflections versus F(θ) and the strain minimized lattice constant (*a*) was obtained from the profile fitting by extrapolating θ to 90° (Y-axis intercept). The calculated lattice constant value for the films deposited on quartz and anode substrate with respect to deposition temperatures are shown in Fig. 3. Thus, the calculated lattice parameter (5.4330 Å) for the film deposited at 500 and 300 °C matches with ICDD card 01-075-0158 for Sm (20 mol%) doped CeO_2_ which indicates the relaxation of strain as compared to the film deposited 100 °C. In general, a decrease in crystallite size lead to the poor structural disorder in the crystal lattice. In the present case of ceria based material, increase in lattice parameter with increasing substrate temperature attributes to the relaxation of pre-existing compressive stress in the nanomaterial, as reported in several literatures^37–39^. S. Mahmud *et al*., attributes this relaxation of strain in doped cerium oxide is due to the increased diffusion of oxygen ion to attain the required stoichiometry^40^. Compared to a quartz substrate, the films deposited over anode substrate shows a relatively higher lattice parameter, which is close to that of bulk indicates the reduction in strain during the growth process of the thin film.

**Figure 4:**
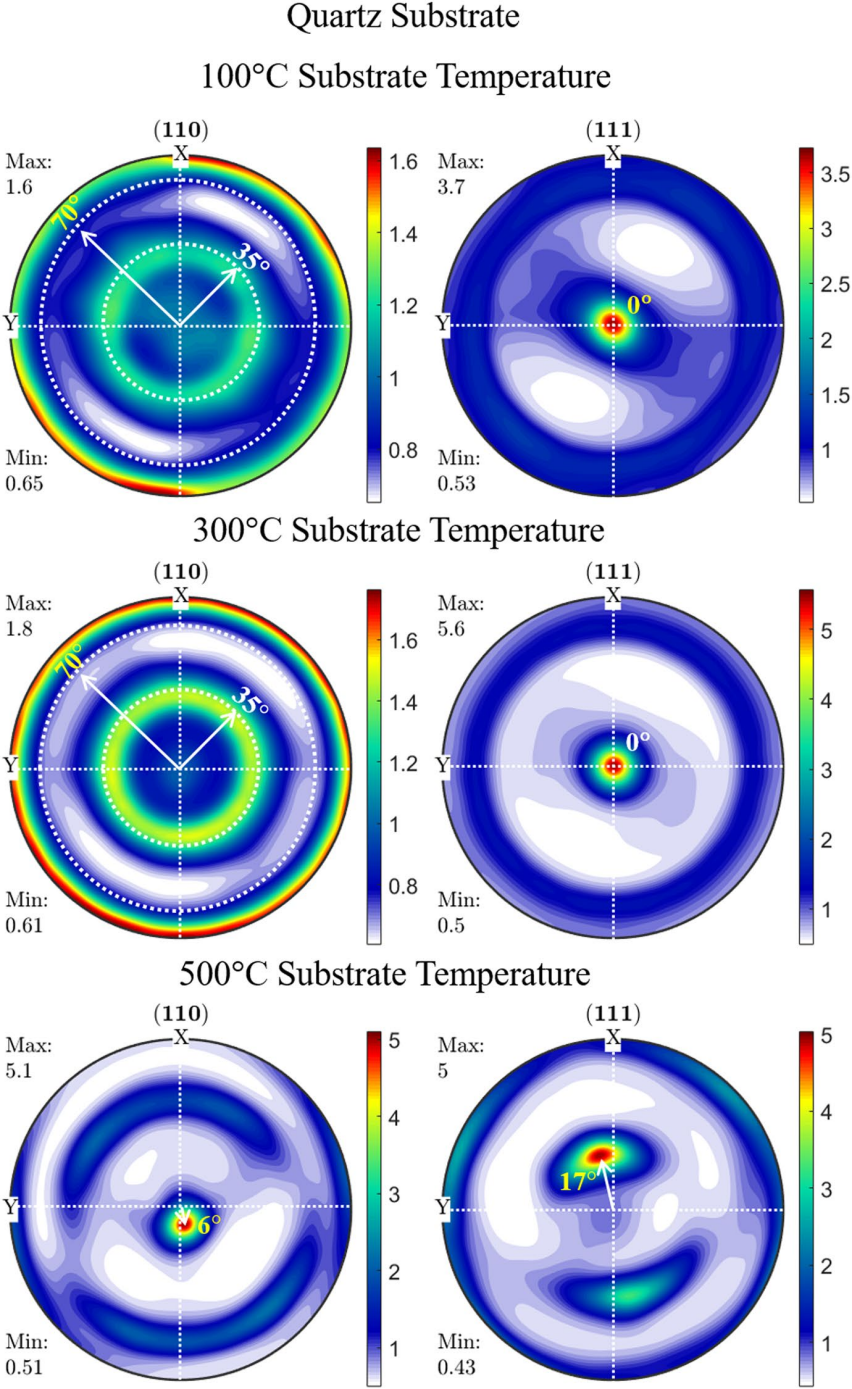
Orientation distribution function extracted from pole figure analysis of SDC film deposited on quartz substrate as a function of substrate temperature.

**Figure 5:**
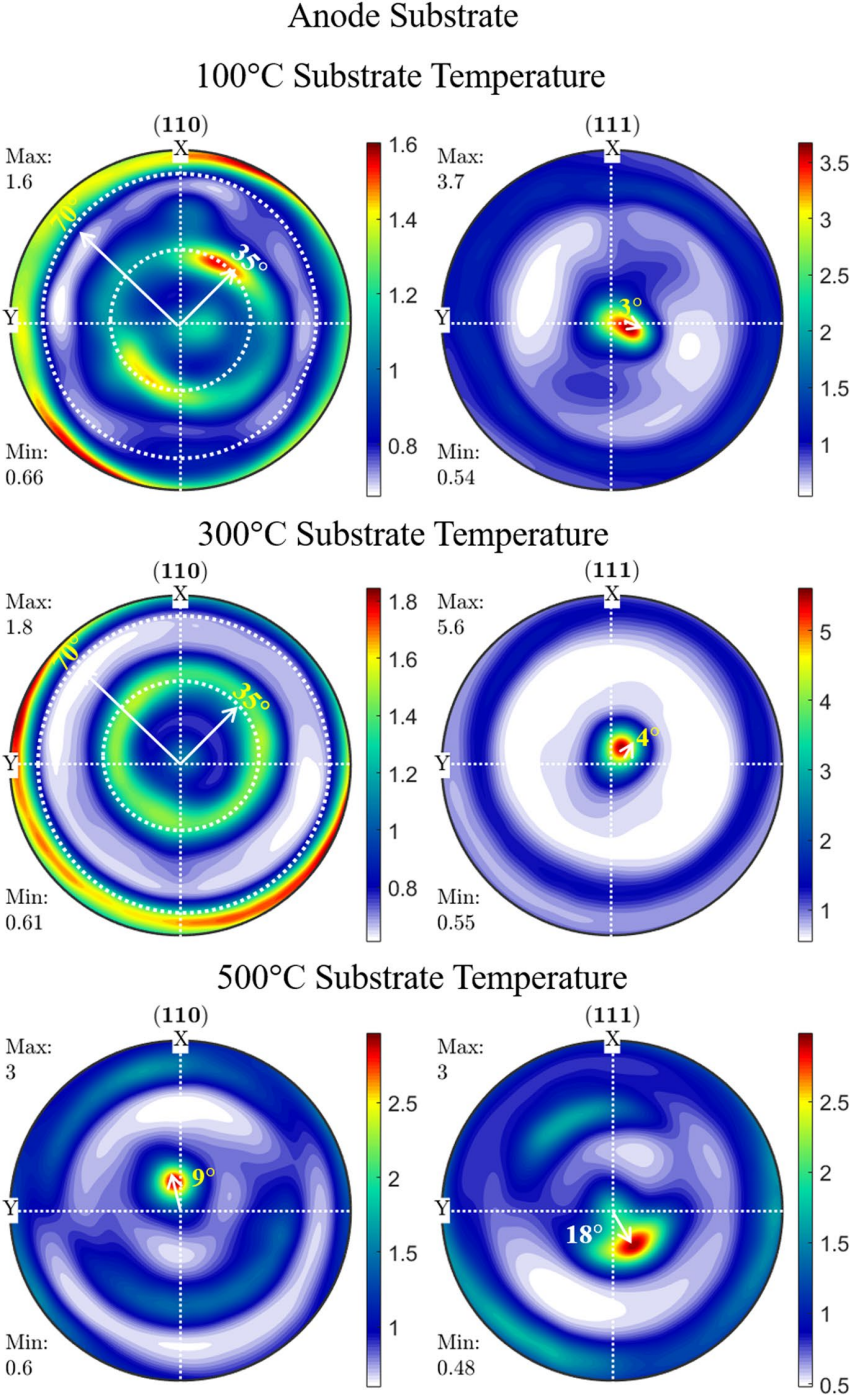
Orientation distribution function extracted from pole figure analysis of SDC film deposited on anode substrate as a function of substrate temperature.

In contrast to the conventional B-B geometry, the pole figure HR-XRD study provides the diffraction information of the crystallographic orientation in both out-of as well as in-plane directions by subjecting the samples at a various azimuthal angle (Ψ & ϕ the angle at fixed 2θ). A sequence of pole figure scans was recorded for the films corresponding to (111) and (220) diffraction peaks. The quantitative data of orientation distribution function (ODF) for a particular (*hkl*) plane was calculated from the pole figure analysis using MTEX program by applying the inversion algorithm method on Matlab platform. In the present work, the FCC crystal with *Fm3m* Laue group was employed with triclinic symmetry as a function of film orientation for the ODF calculation. Figures 4 and 5 shows the ODF information extracted from the pole figure using MTEX^41^ for the (111) and (220) diffraction peaks of SDC films deposited on quartz and anode substrate.

ODF has been represented by the color bar with the logarithmic scale adjusted to its maximum intensity denoting the density of orientation in multiples of a random distribution (m.r.d) around the azimuthal angle rotation. The classification of texture is based on the distribution of ODF value around the pole figure. In general, polycrystalline films exhibit a homogenous distribution of diffracted intensity across the pole figure in all azimuthal angle representing the formation of random texture. However, in the case of preferential orientation, the plane parallel to the substrate concentrate at the center of the pole figure (Ψ = 0) or exhibits a ring pattern at a tilt angle (Ψ = interplanar angle) of that particular (*hkl*) plane in out-of-plane direction denoting the formation of fiber texture. In the biaxial textured thin film, the ring pattern of diffracted intensity converted into a spot at a defined Ψ & ϕ angle across the pole figure. All ODF in the Figs 4 and 5 are represented in the Z-axis direction normal to the thin film surface (Ψ = 0 & ϕ = 0), while the X- and Y- axis facing towards the rolling and transverse direction, respectively, covering around Ψ = 0 to 90° & ϕ = 0 to 360°. It can be seen that the ODF of (111) plane in SDC film deposited at 100 and 300 °C exhibits a maximum m.r.d (represented in the color bar) value of 3.7 and 5.6, respectively, in all azimuthal angles. On the other hand, (220) exhibits a reduced m.r.d value of ~1.6 due to the growth process of films deposited at 100 and 300 °C that favors the formation of (111) plane in comparison to other planes. The film deposited at 100 and 300 °C on quartz substrate exhibits a concentric (111) reflection plane at the center of origin (Ψ = 0°) in the pole figure, which indicates that the (111) plane is almost parallel to the substrate. However, on the anode substrate, (111) plane shows a maximum ODF value concentrated away from the origin by 3 to 4° as the (111) planes are not perfectly parallel to the substrate surface. This off-axis tilt of (111) plane normal to the anode substrate arises due to the increased surface roughness, which leads to the change in surface energy at the interface during the growth process of the thin film^42^. Also, ODF value of (220) plane was not located at the origin but shows the maximum ODF around Ψ = ~35° and ~70° for the entire radial distribution of ϕ = 0 to 360°. These rings belong to the family of (111) reflection plane with respect to the interplanar angle between the (111) and (220) plane in the cubic system which confirms the (220) plane is not parallel to the substrate surface. The film deposited at 300 °Cover both the substrate shows the maximum m.r.d value of 5.6 corresponding to the (111) plane which is higher than the films deposited at 100 °C (m.r.d = 3.7). Thus the film deposited at 300 °C shows a higher degree of (111) preferential orientation on both the substrates than the film deposited at 100 °C. Thus, the ODF analysis represents the clear experimental evidence for the presence of fiber texture associated with the (111) plane of the film deposited on quartz and anode at 100 and 300 °C substrate temperature.

In the case of the film deposited at 500 °C, the ODF of (220) plane shows an interesting effect as compared to the other samples. The (220) diffraction are concentrated at the center of the ODF with an off-axis tilt around Ψ = 6° and 9° on quartz and anode substrates, respectively. In addition to the (220) peak, the (111) peak also shows the maximum ODF value at the origin (Ψ = 0°) of the pole figure with an off-axis tilt around Ψ ~ 18°. Thus the (220) peak was found to be prominent along with the (111) peak as observed in the B-B geometry XRD scan. The tilted concentric circle away from the center of the pole figure with equivalent m.r.d value of (111) and (220) plane signifies the occurrence of texturing competition during the growth process at 500 °C on both the substrates (growth mechanism discussed in section 3.3).

### Surface Morphology of the films

**Figure 6:**
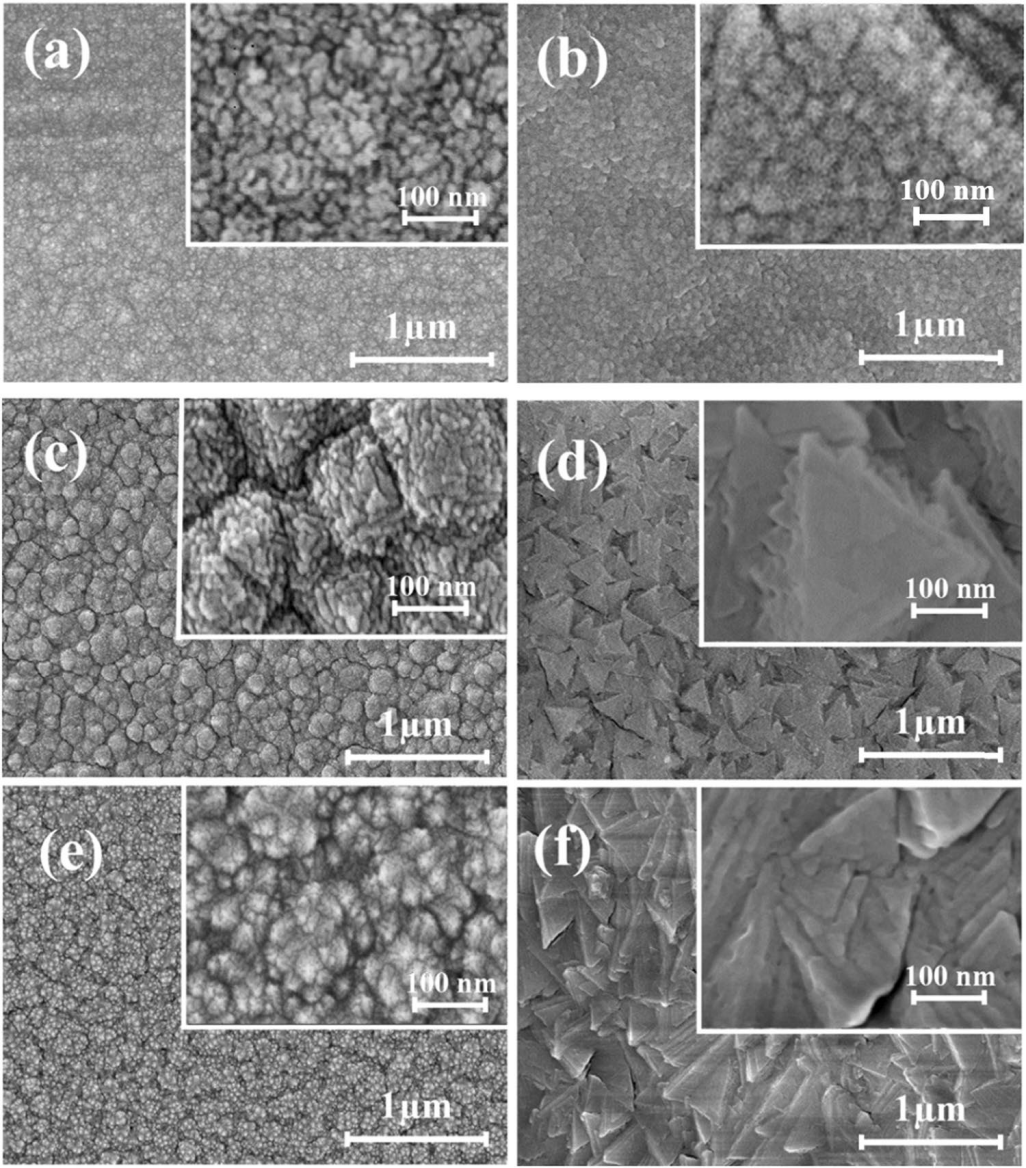
SEM Surface morphology of thin film with low and high magnification at the temperatures of 100 °C (**a**,**b**), 300 °C (**c**,**d**) and 500 °C (**e**,**f**) grown over quartz (**a**,**c**,**e**) and anode (**b**,**d**,**f**) substrates.

Figure 6 shows the SEM surface topography of the SDC film deposited on quartz and anode substrate at various deposition temperatures. The micrographs clearly show the change in surface topography in response to deposition temperature and nature of the substrate. At low substrate temperature (100 °C), both low and high magnification (Fig. 6(a) and (b)) SEM images show the formation of fine granular grains on quartz and anode substrate. On Comparison between the high magnification SEM images of (a) and (b) in Fig. 6, it can be seen that the film deposited on the anode substrate shows well-defined granular shape with higher grain size than the film deposited on quartz. The higher grain size of the film deposited over anode can be attributed to the higher nucleation process of the crystalline island than that of quartz.

SDC films deposited on quartz at 300 and 500 °C substrate temperature showed a round granular like structure at low magnification SEM image. However, at higher magnification (Fig. 6(c) and (e)), triangle like facets structure could be observed inside the round granular grains of the film deposited over a quartz substrate. Unlike the quartz substrate, the films deposited on anode exhibits a faceted structure even at low magnification. The surface morphology of SDC film (Fig. 6(d) and (f)) deposited on anode at 300 and 500 °C exhibits a regular and irregular triangle shaped crystalline facets, respectively. This resemblance of facets structure of thin film at low magnification image denotes the ease of formation of the crystalline island on anode substrate than over quartz.

**Figure 7:**
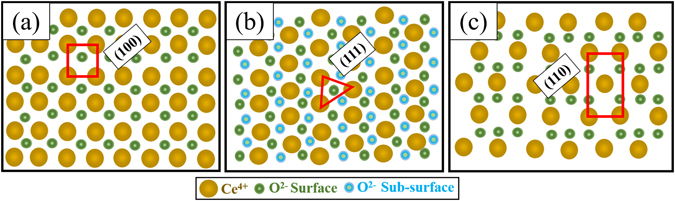
Representation of 2D confinement of (100), (111) and (110) planes of CeO_2_ in cubic fluorite crystal structure.

To understand the CeO_2_ faceted planes in 2D confinement, representative (100), (111) and (110) planes are shown in Fig. 7. At 100 °C substrate temperature, the observed fine spherical grain on both the substrate with miscible faceted structure indicates the reduced degree of preferential orientation. However, the films deposited at 300 °C on both the substrates exhibit a triangle shaped crystalline facets, which matches with the 2D confinement of (111) crystalline plane. This denotes that the film deposited at 300 °C shows the presence of pronounced facets belonging to the (111) grain orientation. At 500 °C substrate temperature, the surface is dominated by the irregular shaped triangle facets due to the co-existence of (111) and (220) planes with mixed triangle and rectangle facets of 2D confinement. Thus, the comparison of surface facets obtained from SEM micrograph with 2D confinement strongly supports the emerging trend in the orientation of thin film observed from the XRD profile, TC value and pole figure analysis.

### Thin Film Growth Mechanism

**Figure 8:**
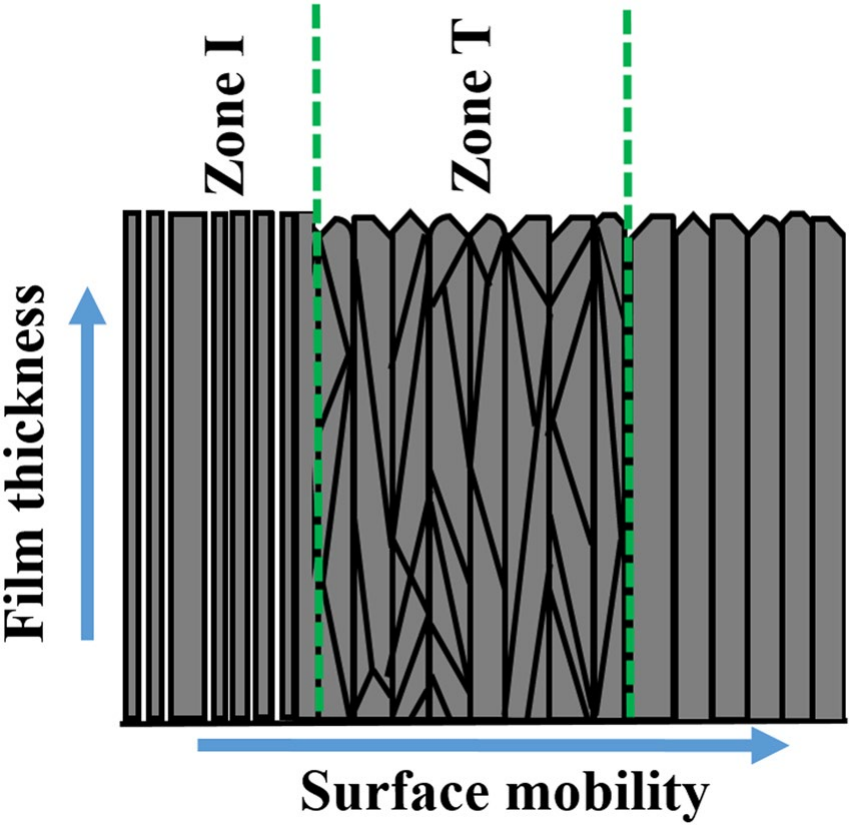
Schematic representation of evolutionary growth mechanism in ESZM model with respect to the surface mobility and film thickness.

Various process parameters such as substrate temperature, deposition rate, base pressure, deposition pressure, the distance between source to the substrate, the thickness of the film and target nature can influence the growth pattern in EB-PVD technique. In the present work, all the process parameters were kept constant, except the substrate temperature in order to correlate its effect on growth mechanism and the corresponding changes in conductivity of the film. The variation in substrate temperature in EB-PVD modifies the quantum of surface diffusion and minimization of energy possessed by the adatom, which plays a significant role in the growth process of the film^43^. In the present work, the coating of ceramic material like ceria in EB-PVD is dominated by the Stranski–Krastanov process, where the incoming adatom initially undergoes two dimension island growth, which gradually transforms into a columnar structure like structure at the later stage^44^. The changes in the substrate temperature result in the variation of the surface diffusion process of arrived adatom, which in turn varies the nucleation process of the island results in either polycrystalline or textured film^30^. The influence of substrate temperature on the crystallographic orientation of thin film can be explained by the extended structure zone model (ESZM) as proposed by Mahieu *et al*.^27^. According to the ESZM model, the growth mechanism of the thin film varies under three different zones, namely Zonal I, T and II (Fig. 8).

Zonal I model assumes the formation of a film with a negligible mobility of adatom to overcome the surface diffusion barrier, which results in randomly arranged crystal habits along the thickness of the film. In Zonal T model, the presumption is that the adatoms have higher energy to overcome the surface diffusion barrier from one grain to another with immobile grain boundary. As a result, the grain growth follows the thermodynamically favorable crystal facets leads to orientation of thin film with a V-shaped column in an evolutionary way as represented in Fig. 8 ^21^. In Zonal II model, the adatoms have sufficiently higher surface mobility, which in turn induce the recrystallization process among the stable grain beyond the grain boundary leading to the further minimization of the surface energy by affecting the fastest growing facets. Accordingly, the surface mobility of adatom in ESZM model defines the crystallographic nature of the deposited film.

The surface mobility of adatom over the substrate in EB-PVD is mainly governed by two parameters, namely, the kinetic energy of evaporated atom from the target and the substrate temperature^45, 46^. The high kinetic energy of electron beam (e-beam) hitting the target gets converted into heat energy leading to the sublimation of the target under high vacuum (>2 × 10^−5^ mbar). The relationship between the kinetic energy of evaporated atom and vaporization temperature is given by Boltzmann temperature (equation 6).

5 $$E=\frac{3}{2}k{T}_{\nu }$$

where, *E* is the kinetic energy of the evaporated adatom, *k* is the Boltzmann constant, *T*_*v*_ is the vaporization temperature. Accordingly, the kinetic energy of the evaporated adatom (*E*) is directly proportional to the vaporization temperature (*T*_*v*_). Thus, an increase in deposition rate or e-beam current, in turn, increases the kinetic/thermal energy of the vaporized atom. Since in present work, the constant deposition rate results in the negligible change of kinetic energy of adatoms (E)^47^. As a result, the variation in substrate temperature dominates the change in surface mobility of adsorbed adatom, which in turn determines the crystallographic nature during the film formation as represented by the homologous temperature (*T*)^48, 49^.

6 $$T=\frac{{T}_{s}}{{T}_{m}}$$

where *T*_*s*_ is the substrate temperature, and *T*_*m*_ is the melting temperature of the depositing material. The melting point of ceria has been reported as 2670 K^50^. Thus, the homologous temperature T was calculated to be 0.14, 0.21 and 0.27 for the films deposited at 100, 300 and 500 °C, respectively. The increase in substrate temperature increases the value of T leading to the higher surface mobility of adatom.

**Figure 9:**
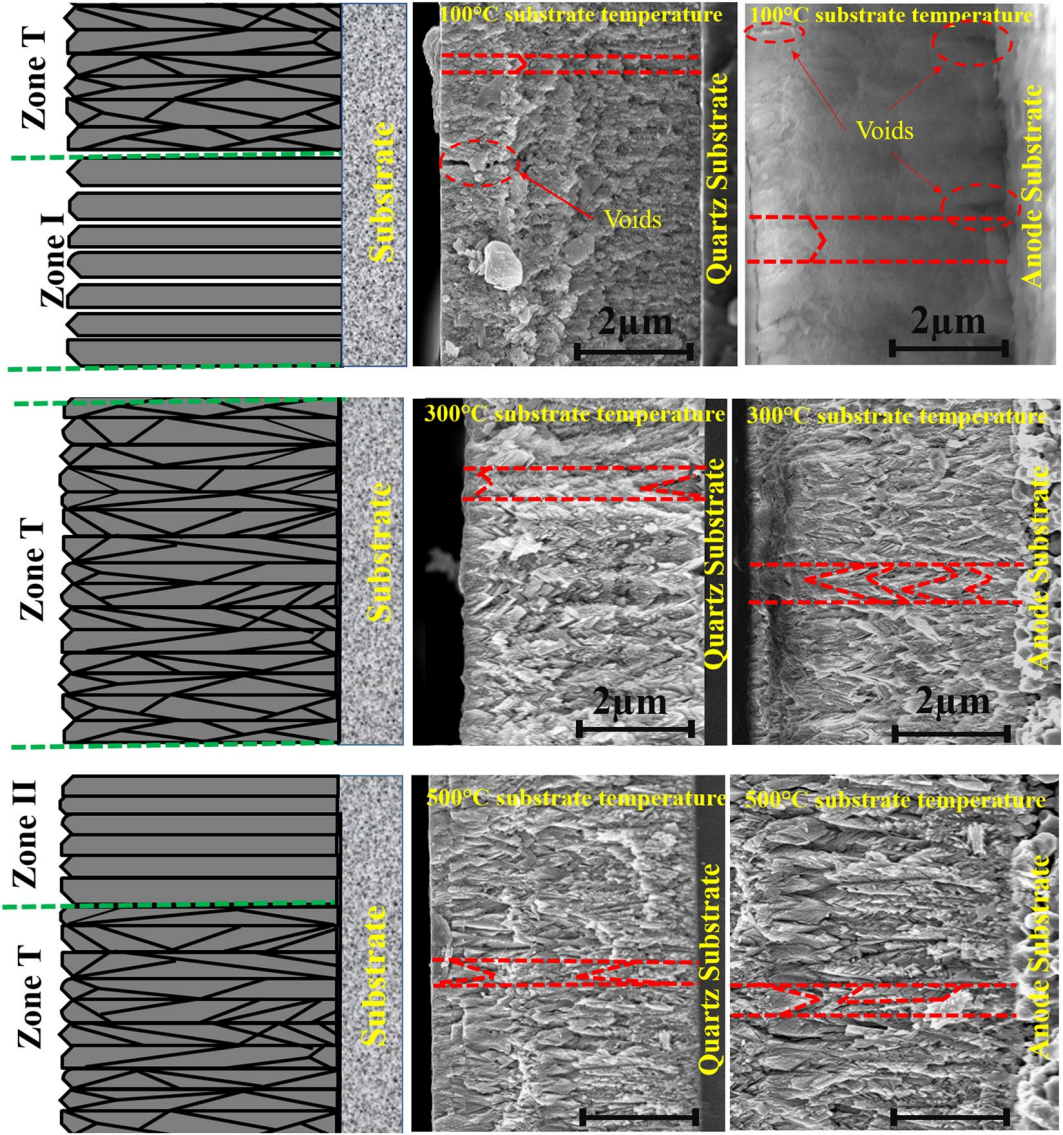
Cross-sectional view of a thin film deposited at 100, 300 and 500 °C substrate temperature correlated with the ESZM model.

Cross-sectional SEM analysis was carried out to understand the growth processes of the thin film. SEM images shown in Fig. 9 indicates the presence of V-shaped columnar structure independent of the substrate temperatures or substrate nature. Thus, the growth mechanism of all the films was expected to follow the regime around Zonal-T model in ESZM. The preferential orientation of the film deposited at 100 and 300 °C indicates the surface mobility of adatom sufficiently reaches its kinetically determined faceted structure as explained in the Zone T. The kinetically determined faceted plane along the perpendicular direction terminates the planes having the lowest growth rate. This competitive growth of neighboring grains indicates the survival of fastest facets with preferential orientation by burying the adjacent grain leading to the V-shaped columnar structure in an evolutionary way (Fig. 9). The growth process of the films in evolutionary mode was proposed by Van der Drift^51^, who suggested that the crystal planes with lower surface energy undergo fastest vertical growth rate in perpendicular direction during the film formation^52^. Fronzi *et al*., used *ab initio* atomistic thermodynamics model using density function theory (DFT) and calculated the surface energy of the low index planes in CeO_2_ for (111), (110) and (100) as 0.037, 0.057 and 0.230 eV, respectively^53^. Thus, the (111) plane undergo fastest growth rate in a perpendicular direction with an increase in film thickness as supported by Van der Drift^51^. The presence of highly preferred (111) orientation and V-shaped columnar structure (Fig. 9) in the film deposited at 100 and 300 °C exemplifies that the growth mechanism falls around the Zonal T regime. However, the results of XRD and pole figure analysis indicates the films deposited at 300 °C on both the substrate poses increased the degree of preferential orientation than 100 °C. This increase in the degree of (111) orientation arises from the higher surface mobility of adatom at 300 °C in comparison to 100 °C. Thus, the film deposited at 300 °C substrate temperature consists of defined V-shaped columnar structure, which is in great agreement with the Zonal T growth mode in ESZM. However, the V- shaped columnar structure was not well defined and separated by voids in the cross-sectional image of the film deposited at 100 °C on both the substrate. These fuzzy V-shaped columns with voids at 100 °C substrate temperature signifies the growth mechanism falls in the regime of Zonal-I in addition to the Zonal T model^54^.

Increase in surface mobility of adatoms at 500 °C lead to the mixed orientation of (110) and (111) planes as observed from the XRD pattern. Thus, the presence of (220) plane along with (111) indicates the adatom mobility provides enough displacement energy to diffuse within the grains and partially beyond the grain boundary resulted in the recrystallization process along the columnar structure. This recrystallization process between the islands partially terminates the competitive evolutionary mode of fastest growing (111) faceted plane leading to the formation of next possible lower surface energy plane of (220). Thus, the growth process at 500 °C results in a thermodynamically textured film with the mixed orientation of (111) and (220) planes. The cross-sectional view of SEM image, exhibits a diminished V-shaped columnar structure along with solid column indicates the utilization of surface mobility of adatom within the grain as well as partially crossing beyond the grain boundary. Hence, the experimental results clearly designate the growth mode of SDC thin film deposited at 500 °C on both the substrates falls in the regime between Zonal T and Zonal II mode of ESZM as indicated in Fig. 9.

### Raman Spectroscopy

**Figure 10:**
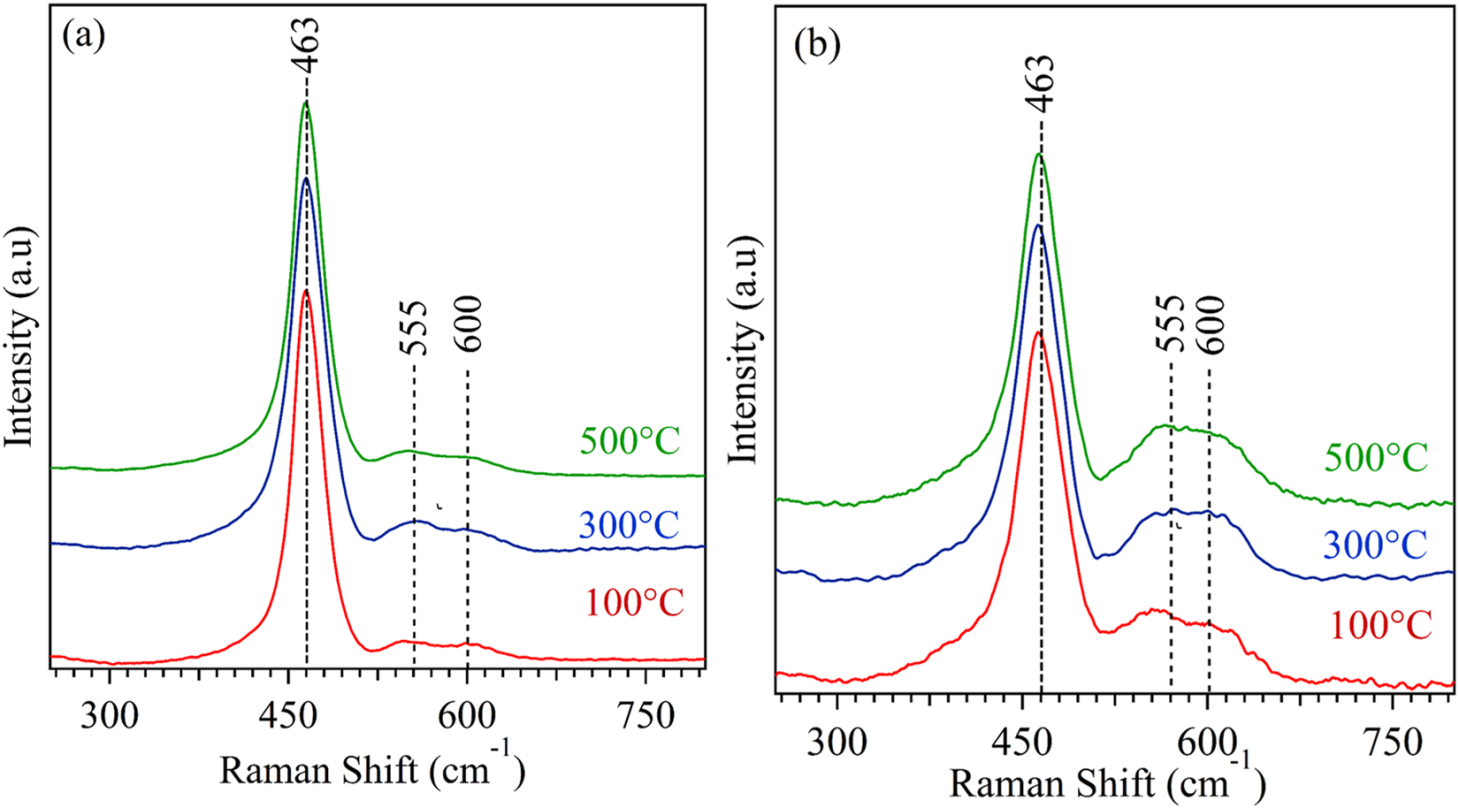
Raman Spectra of SDC thin film deposited at 100, 300 and 500 °C substrate temperature.

Raman spectroscopy studies were carried out to investigate study the influence of crystallographic orientation on defect chemistry of thin film with respect to substrate temperature. Figure 10 shows the Raman spectra of SDC films as a function of substrate temperature on quartz and anode substrate. Bulk CeO_2_ consists of an intense band of Raman spectra at 465 cm^−1^ corresponding to the F_2g_ vibrational mode in octahedral Ce-O8^55^. In the present case, an intense Raman peak around ~463 cm^−1^ was observed from F_2g_ vibrational mode of crystalline CeO_2_. The observed F_2g_ bands at 463 cm^−1^ for the film were found to be downshifting by 2 cm^−1^ as compared to the bulk CeO_2_. This downshifting of the F_2g_ band in SDC film corresponds to the lattice expansion induced by the doping of Sm^3+^ inside the ceria lattice^56^. Weak diffusive Raman spectra observed in the range of 500–650 cm^−1^ arise from the overlapping of two bands at 555 and 600 cm^−1^ corresponds to the oxygen vacancy induced by the Sm^3+^ doping and Ce^3+^, respectively.

The *F*_*2g*_ vibrational mode of Ce-O8 in SDC thin film is very sensitive to the lattice distortion induced by the absence/presence of oxygen vacancy. Thus, the observed broadness and asymmetry nature of F_2g_ band at ~463 cm^−1^ designates the non-stoichiometric nature in the CeO_2_ lattice^57^. Spatial correlation model can be used to calculate the oxygen vacancy concentration from the *F*_*2g*_ band of ceria using the following equation^58^.

7 $$N=\,\frac{3}{4{\rm{\pi }}{L}^{3}}$$

where, *N* is the defect concentration (cm^−3^), and *L* is the correlation length between two oxygen vacancy defects. Table 1 shows the calculated defect concentration for the films. Irrespective of the substrate temperature, a marginal change in oxygen vacancy concentration was observed for the films deposited over a particular substrate. However, the films deposited over the anode substrate showed higher vacancy concentration than one on the quartz substrate, which can be attributed to the effect of substrate peak overlapping with the SDC film. As a result, the Raman spectroscopic study emphasizes a negligible influence of substrate temperature on defect concentration of the deposited films.

**Table 1:** Calculated oxygen vacancy concentration using spatial correlation model for the thin film with respect to substrate temperature.

Substrate Temperature (°C)	Oxygen vacancy concentration (×10^21^ cm^−3^)	
	Quartz Substrate	Anode Substrate
100	1.5	2.7
300	1.6	2.8
500	1.8	2.5

### Effect of texturing on oxygen ion transport

**Figure 11:**
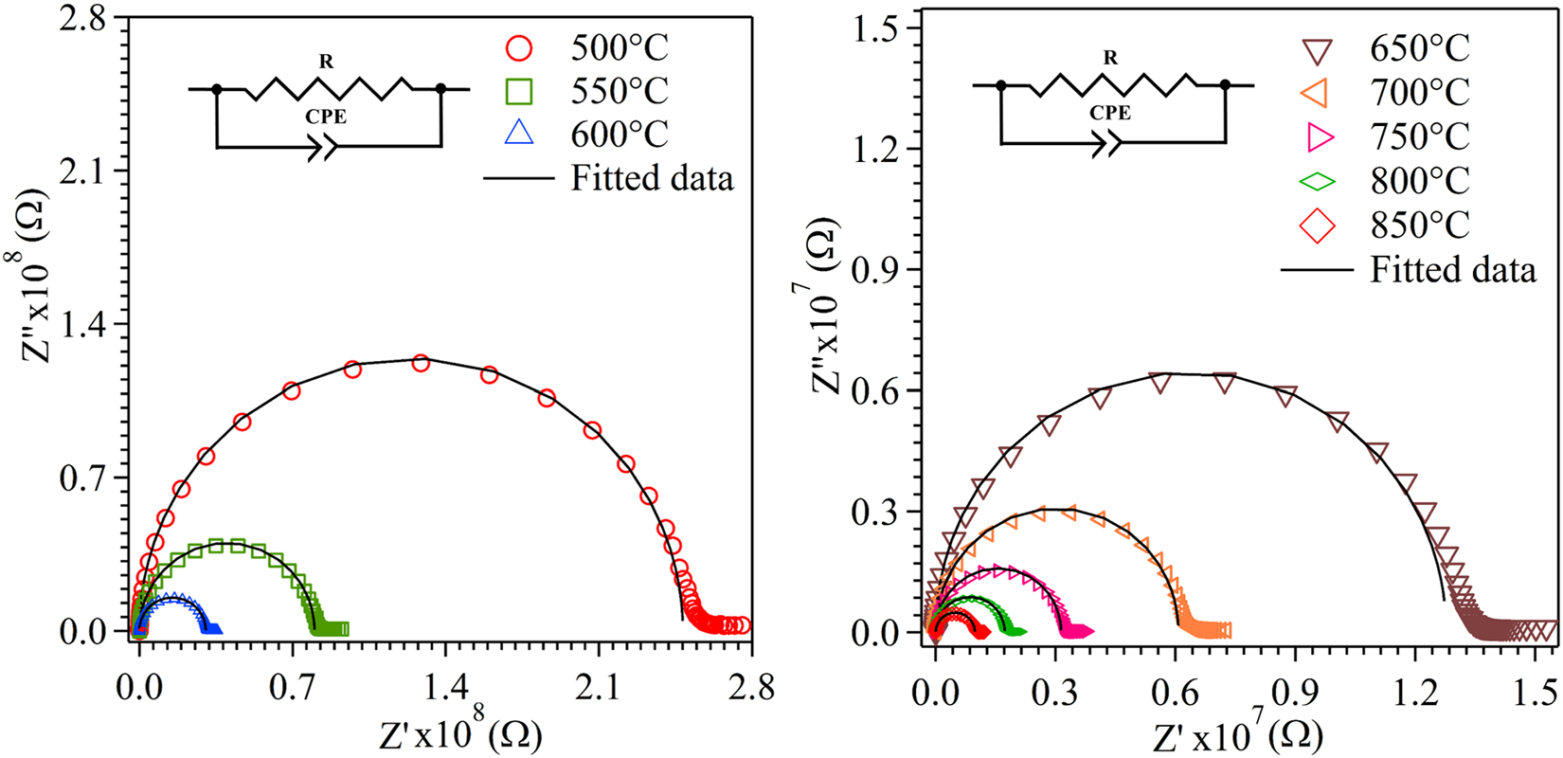
Nyquist plot measured at the temperature range from 500 to 850  °C for the thin film deposited at 100 °C substrate temperature.

Impedance spectroscopy measurement was performed to evaluate the ionic conductivity and activation energy of the films with respect to crystallographic orientation. The conductivity of bare quartz and anode substrates (without SDC film) were tested at high temperature. The quartz substrate showed negligible electrical conductivity as compared to the quartz substrate having SDC film due to its large electrical resistivity. On the other hand, the anode substrate showed higher electrical conductivity, equivalent to that of SDC film, which denotes the contribution of conductivity from SDC and NiO material present in the anode substrate. Hence, the impedance measurement was performed only for the film deposited on a quartz substrate, in order to elucidate the electrical conductivity of SDC film alone with negligible substrate contribution. Figure 11 shows the temperature-dependent Nyquist plot of the film deposited at 100 °C. The resistivity of the film decreased with the increase in testing temperature from 500 to 850 °C was due to the enhancement of oxide migration by the thermal activation process.

In general, the Nyquist plot consists of three distinct arcs corresponding to the contribution of grain, grain boundary and electrode processes. The distinct arcs are due to the difference in time constants during the migration of oxide ion along the grain (g), grain boundary (gb) and electrode interface. According to the brick layer model, the resistance of the in-plane impedance measurement across the thin film can be calculated using the below equations^59^.

8 $${R}_{gb}=\,\frac{\,{\rho }_{gb}{\delta }_{gb}{N}_{gb}}{A}$$

9 $${R}_{g}=\,\frac{\,{\rho }_{g}L\,}{A}$$

10 $${N}_{gb}=\frac{L}{{d}_{gb}}$$

where R_g_ and R_gb_ is the grain and grain boundary resistance, N_gb_ is the number of grain boundary across the samples, ρ_g_ and ρ_gb_ is the specific grain and grain boundary resistivity, L is the inner electrode distance, A is the cross-sectional area of the thin film, d_gb_ is the average grain size and δ_gb_ is the grain boundary thickness.

**Figure 12:**
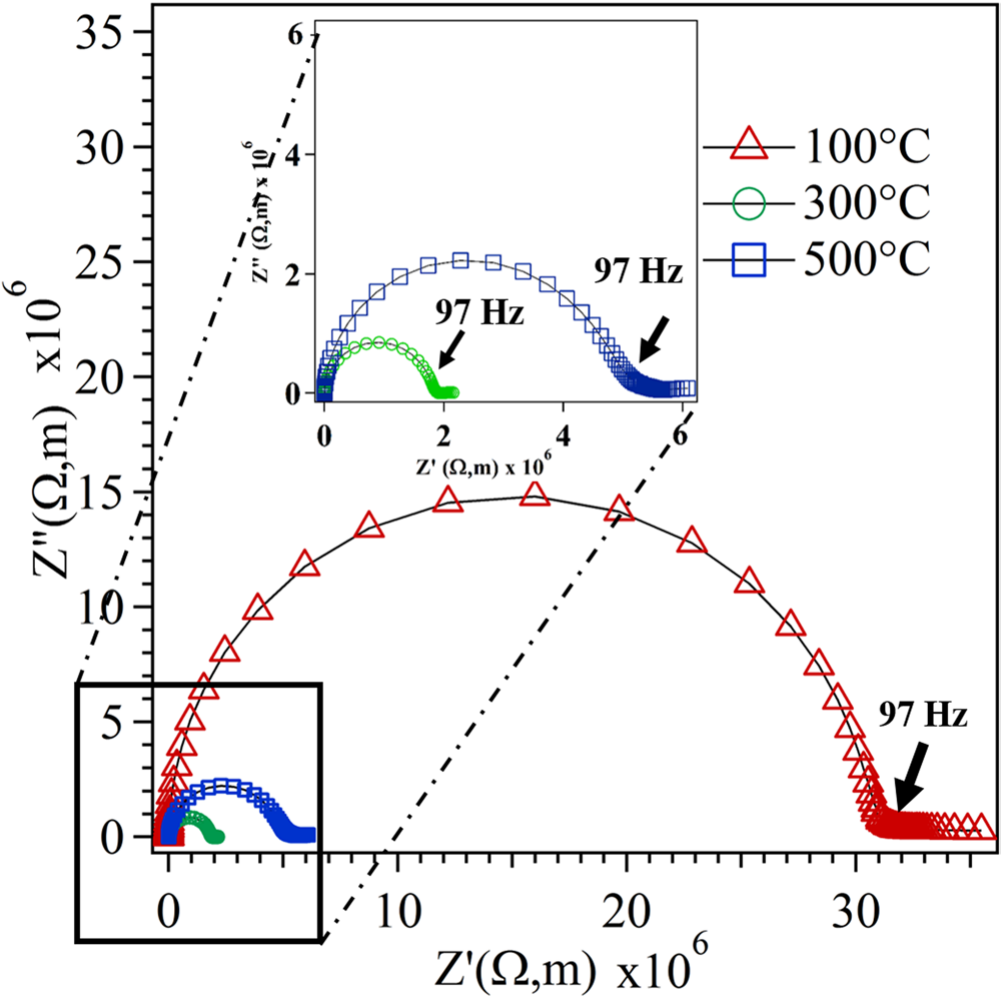
Complex impedance plot of all thin films measured at 600 °C in air atmosphere.

The capacitance of thin film can be calculated from the impedance measurement according to the following equation

11 $${C}_{gb}=\,\frac{{\varepsilon }_{0}\,{\varepsilon }_{r}\,A}{{\delta }_{gb}\,{N}_{gb}}\,=\frac{{\varepsilon }_{0}{\varepsilon }_{r}\,A\,{d}_{gb}}{{\delta }_{gb}{\rm{L}}}$$

12 $${C}_{g}=\,\frac{{\varepsilon }_{0}{\varepsilon }_{r}\,A\,}{L}$$

where C_g_ and C_gb_ is the capacitance of grain and grain boundary, ε_o_ is the permittivity of the vacuum, ε_r_ is the dielectric constant of the material. The dielectric constant of the CeO_2_ based electrolyte is ε_r_ = ~30^60^. In the present work, the sample geometry L = 1.5 cm and A = 5.9 × 10^−6^ cm^2^. The grain and grain boundary in nanometre scale is assumed to be in the range of 10 to 100 for the ratio of d_g_/δ_gb_. The calculated capacitance value of grain (C_g_) is 10^−15^ F and grain boundary (C_gb_) is 10^−14^ to 10^−13^ F, respectively. The calculated capacitance reported in the present work is for nanocrystalline thin film based ceria system. On the other hand, Rai *et al*.^61^ reported the electrical properties of pellet based bulk ceramic with an across-plane configuration in doped cerium oxide system that exhibits the geometrical factor (A/L) of electrodes by ~16.7 cm, which is much greater than the values observed in the present work ~4 × 10^−5^ cm. In this regard, the higher grain size combined with higher A/L will increase the capacitance of grain and grain boundary in the pellet based system as compared to thin film^62^. Hence, the capacitance value obtained from the across-plane configuration of pellets based bulk system is not comparable with the in-plane configuration of the thin film as measured in the present case However, the experimental setup introduces a stray capacitance value of about 10^−12^ F, which is several order of magnitude higher than the capacitance value of grain and grain boundary of the film^63^. High stray capacitance value in the film makes it impossible to separate the resistance contribution between grain (R_g_) and grain boundary (R_gb_) during impedance measurement. Thus, the measured resistance from Nyquist plot arises from the summation of both grain and grain boundary contribution. In accordance, the equivalent circuit model with single parallel RC element was used to fit the experimentally absorbed impedance plot (Fig. 11), where the ideal capacitor (C) was replaced using the constant phase element (CPE) for the slightly depressed arc of the Nyquist plot^46^. The estimated stray capacitance using the equivalent circuit model for the SDC films was in the range around 2.4 −7.5 × 10^−12^ F. In general, the relaxation frequencies of grain (*f*_*g*_ = (2πR_g_C_g_)^−1^) and the grain boundary (*f*_*gb*_ = (2πR_gb_C_gb_)^−1^) are independent of geometrical factor. However, at high temperature (>~450 °C) the relaxation frequency of both grain and grain boundary material tend be too large to separate the individual contributions of grain and grain boundary in the Nyquist plot^62^. In the present case, the Nyquist plot of thin film measured in the range of 450 to 850 °C resulted in a single arc which arise due to total conductivity (Grain + Grain Boundary). Moreover, the substrate/ experimental setup^64^ for in-plane configuration in present thin film introduce a stray capacitance in the order of 10^−12^ F, which is several order of magnitude higher than the typical capacitance value of grain and grain boundary of the film^63^. Thus, high stray capacitance value observed in the in-plane configuration of the film makes it impossible to separate the contribution between grain (R_g_) and grain boundary (R_gb_) resistance, which effectively reduces the Nyquist plot to a single arc as shown in Fig. 12. Many reports are availoable which has pointed out the presence of stray capacitance in the in-plane configuration of ceria based thin film, which is in good agreement with the present work^65^. In addition, Navickas *et al*., experimentally carried out the simultaneous in- and across-plane conductivity measurement in the YSZ thin film and observed a single semicircle (Nyquist plot) in the in-plane configuration due to the presence of unavoidable stray capacitance from the substrate/experimental setup^66^. Thus, the in-plane configuration in the present work expected to increase the contribution of grain boundaries, since in the EIS measurement the current line passes through the columnar grain structure. This demonstrates that the total contribution on the conductivity of samples consists of both grain and grain boundary. Our results are in good agreement with the previously reported data for the oriented film in doped cerium oxide^24, 67^.

The resistance value obtained with best fitting results were normalized and correspondingly converted into conductivity using the relation

13 $$R=\rho \frac{{\rm{L}}}{{\rm{A}}}$$

14 $$\sigma =\frac{1}{\rho }$$

where, R is the experimental resistance of the thin film, ρ (Rg + R_gb_) is the specific resistivity of the deposited material and σ is the conductivity of the sample. For comparison, the normalized Nyquist plot measured at 600°C for the film deposited at different substrate temperature are shown in Fig. 12. It can be seen from the Fig. 12 that the resistivity of the SDC film decreases in the order of 300 °C < 500 °C < 100 °C substrate temperature.

**Figure 13:**
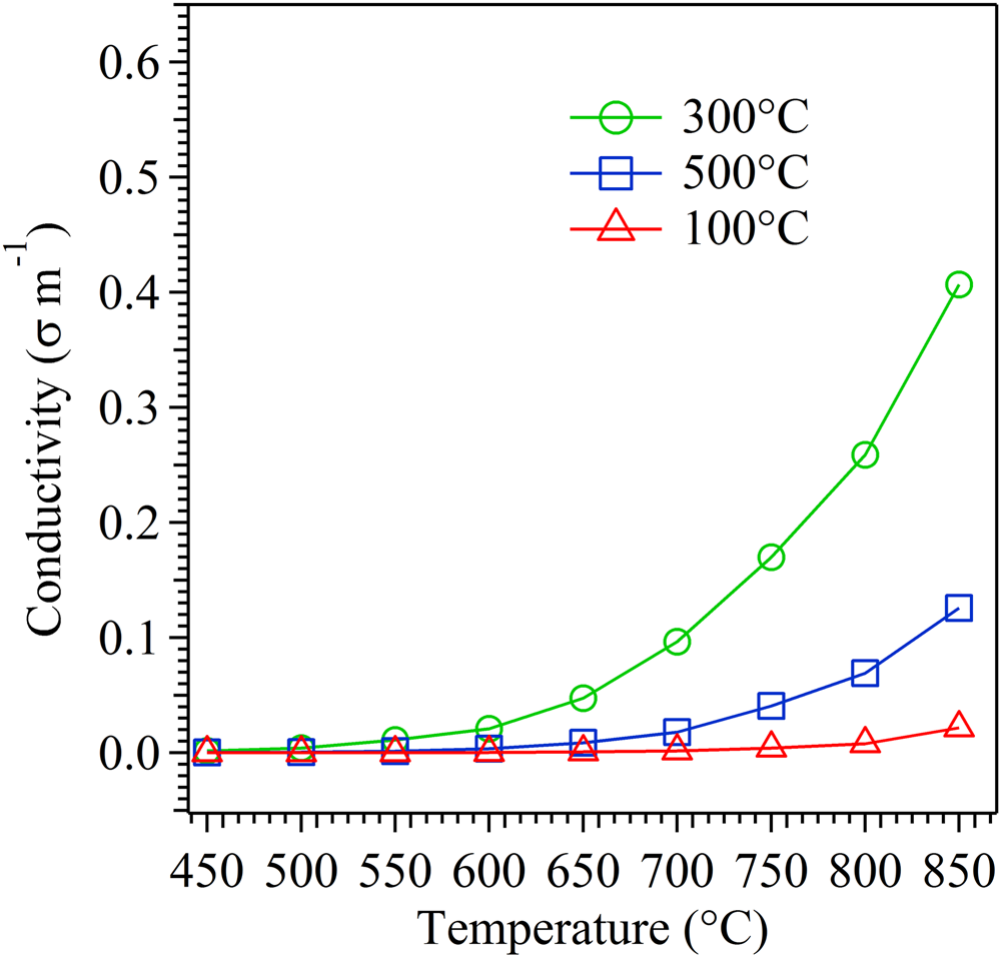
Conductivity of SDC thin film electrolyte as a function of temperature with respect to the substrate temperature.

Figure 13 shows the temperature-dependent conductivity data for the SDC thin film deposited at 100, 300 and 500 °C. The total conductivity of the films exponential increases with increase in temperature attributed to the thermal activation process on conduction mechanism. The film deposited at 300 °C shows a maximum enhancement in conductivity, followed by 500 and 100 °C deposition temperature.

The activation energy of SDC film with respect the total conductivity was calculated using Arrhenius equation 17.

15 $${\rm{\sigma }}{\rm{T}}={\rm{A}}\,\exp (-\frac{{\rm{E}}}{{\rm{kT}}}\,)$$

where, E is the activation energy, k is the Boltzmann constant, T is the temperature, and A is the pre-exponential factor.

**Figure 14:**
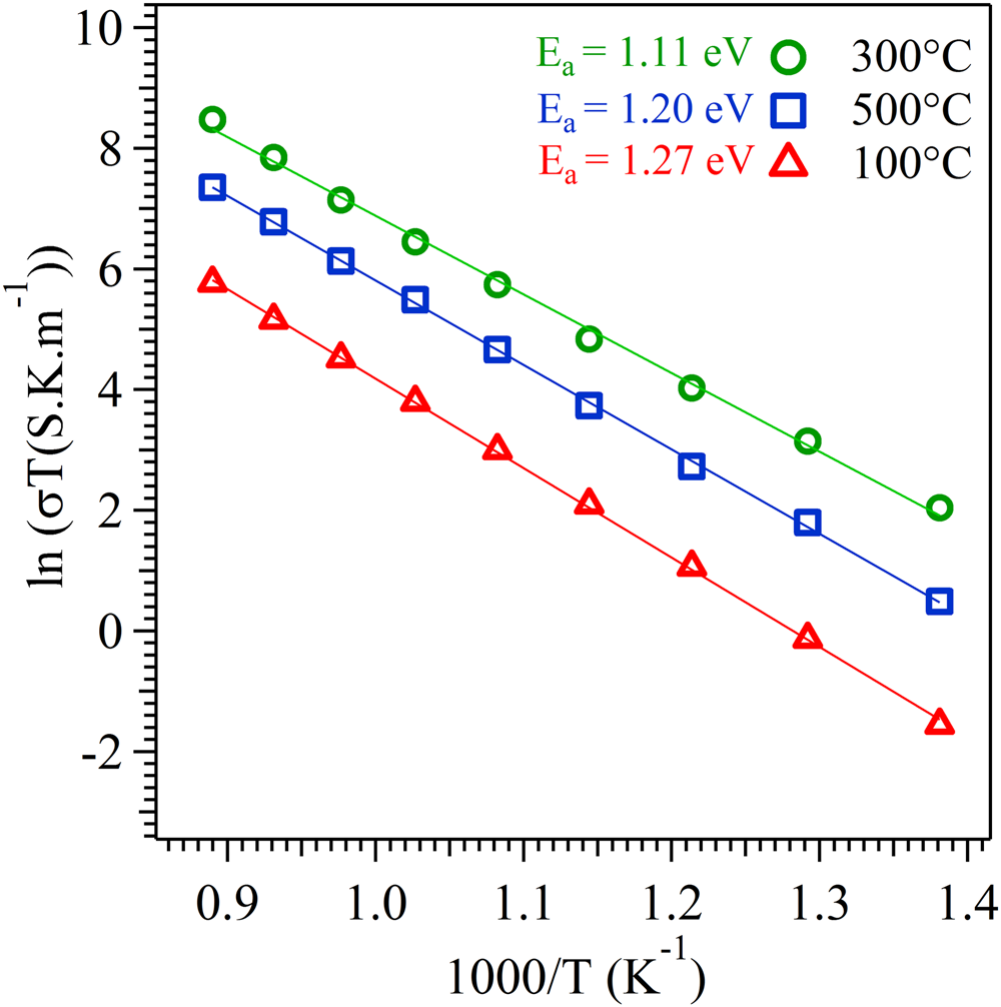
Arrhenius plot for a thin film with respect to the substrate temperature.

Figure 14 shows the Arrhenius plot of the SDC film deposited at different substrate temperature. The activation energy *E* calculated from Arrhenius equation was found to be 1.27, 1.11 and 1.20 eV for the SDC film deposited at 100, 300 and 500 °C, respectively. The variation in total conductivity of the SDC film deposited at different substrate temperature and their associated activation energy, suggest that the mechanistic aspect of conductivity considerably depends on the magnitude of preferential orientation^68^. The increase in the preferential orientation of the film, in turn, decreases the anisotropy nature and density of the grain boundary (GB) in the out-off plane direction^69^. Obviously, the total conductivity of the film substantially decreases with increases in grain boundary area due to the depletion of oxygen vacancy by the negative space-charge layer enriched through trivalent dopant (Sm^3+^) in cerium oxide^70^. In this regard, the increase in the preferential orientation of film will enhance the overall ionic conductivity by reducing the grain boundary resistance^71^. Similar behavior was observed in the various literature reported for the trivalent doped cerium oxide thin film^64, 72^.

XRD pattern, TC value, pole figure and SEM analysis indicate that the film deposited at 300 °C possess a higher degree of preferential orientation in (111) direction as compared to the other films. This predominant (111) orientation at 300 °C results in highest ionic conductivity with reduced activation energy. From the pole figure analysis, it is known that the film deposited at 300 °C possess maximum ODF value (m.r.d = 5.6) for (111) plane results in a reduced anisotropy nature and density of grain boundary area in out-off plane direction^52^. This crucial aspect of reduced grain boundary effect facilitates the easy hopping of oxygen ion through the film with a lower activation energy (1.11 eV). It is evident from the impedance results, at a higher temperature (850 °C), the activation energy of the film deposited at 300 °C possess, almost 7.7 and 13.4% larger than the film deposited at 500 and 100 °C respectively.

After 300 °C, the ionic conductivity follows the order of thin film deposited at 500 and 100 °C in decreasing sequence of conductivity. The film deposited at 500 °C exhibits equivalent ODF value for (111) and (220) plane in pole figure indicates the presence of mixed orientation. In addition to this, the cross-sectional view of SEM image clearly exhibits the growth mechanism of the film at 500 °C lying between the Zonal T and II modes of ESZM. Obviously, this nature of growth process with mixed orientation results in increased anisotropy nature and the density of the grain boundary, which augments the barrier during hopping process of oxygen ion. Accordingly, the film deposited at 500 °C results in lower conductivity with increased activation energy (1.20 eV) than the film deposited at 300 °C.

The preferential orientation of (111) plane is more prominent in the film deposited at 100 °C as compared to the film deposited at 500 °C. However, the film deposited at 500 °C shows increased ionic conductivity as compared to 100 °C. This lower ionic conductivity at 100 °C attributes to the presence of voids along the thickness (column) of the film as its growth mechanism partially falls under the Zonal I mode in addition to the Zonal T as represented by the cross-sectional view of SEM image. The presence of voids in the SDC film at a lower substrate temperature (100 °C) in tune leads to the poor contact between columnar grains, resulting in an increased activation energy (1.27 eV) with lower conductivity in comparison to the other films. Therefore, the ionic conductivity of the thin film is considerably affected by the crystallographic orientation of thin film deposited on quartz at various substrate temperature. As SDC film follows the similar trend in the growth mechanism on both the substrate, the change in conductivity of the flim over quartz substrate was nearly comparable to the film deposited over anode substrate. Thus, the predominant (111) orientation of thin film grown corresponding to Zonal T model of ESZM exhibits reduced grain boundary factor, which is expected to increases the overall ionic conductivity of the film deposited on both the substrates.

## Conclusion

In summary, we have developed a preferentially oriented SDC film on anode and quartz substrate by tuning the deposition temperature in EB-PVD technique. The growth mechanism of oriented thin film demonstrated via extended structural zonal model using the cross section view of SEM image. SDC film deposited at 300 °C on both the substrates showed highly preferred (111) orientation as compared to the film deposited at 100 and 500 °C. The optimum surface mobility of adatom at 300 °C favored the formation of (111) faceted plane in out-off plane direction while remarkably decreasing the anisotropic nature and grain boundary area, thereby endowing the easy hopping of oxygen ion during the conduction process. The impedance measurement showed a superior ionic conductivity (3.3 × 10^−1^ S m^−1^ at 650 °C) for highly orientated thin film obtained at 300 °C followed by 500 °C (1.1 × 10^−1^ S m^−1^) and 100 °C (0.2 × 10^−1^ S m^−1^). Thus, the ionic conductivity measurement carried out in thin film increases with increase in the degree of preferential orientation. Hence a profound understanding is necessary for controlling the fundamental properties of orientation in a thin film with respect to processing, in order to tailor the electrolyte materials for ITSOFC application.

## Methods

### Electrolyte preparation

Samarium (20 mol%) doped cerium oxide (SDC) was prepared from cerium nitrate hexahydrate (Ce(NO_3_)_3_.6H_2_O, Himedia, 98%) and samarium nitrate hexahydrate (Sm(NO_3_)_3_.6H_2_O, Alfa Aesar, 99.9%) by precipitation method. Briefly, cerium and samarium nitrate precursors were dissolved in 100 ml of deionized water at required stoichiometry, and 1 N of ammonium hydroxide (Merck, 30%) solution was added dropwise in order to maintain the pH ~9–10. After 4 hours of continuous stirring, a pale yellow precipitate was obtained and subsequent drying at 80 °C overnight resulted in SDC powder. Pellet made by the compaction of powder using 5 tons isostatic press was calcined at 800 °C for four hours and used as a target in an EB-PVD deposition.

### Anode Preparation

NiO-SDC (60:40 wt%) nanocomposite material was synthesized using co-precipitation method. The required quantity of the as-synthesized SDC powder was dispersed in 100 ml of deionized water using ultrasonication technique. A 200 ml (0.16 M) nickel nitrate hexahydrate (Ni(NO_3_)_3_.6H_2_O, Himedia, 98%) of deionized water was added dropwise to the dispersed SDC solution under vigorous stirring. Subsequently, 1N ammonium hydroxide (Merck, 30%) solution was added dropwise into the mixture while maintaining the pH around 9–10 for precipitation. A pale green color precipitate obtained after stirring for 4 hours was washed and dried at 100 °C to obtain SDC-NiO (40:60 wt%) nanocomposite powder. The pellets obtained by isotactic hydraulic pressing at 10 tons of pressure were then sintered at 1200 °C for 4 hours and used as anode substrate for electrolyte deposition in EB-PVD technique.

### Deposition of Electrolyte thin film

Quartz (Ant labs, 99.99%, amorphous) and anode (as detailed in section 2.2, polycrystalline) were used as a substrate for the deposition of SDC thin film in EB-PVD method (BC-300 Vacuum box coater, Hind High Vacuum, India). The base pressure was maintained at ~1 × 10^−6^ mbar while keeping the deposition pressure at ~2 × 10^−5^ mbar. The deposition rate was kept constant at ~1 Å/s while varying the substrate temperatures from 100, 300 to 500 °C. The thickness of the SDC film was maintained around 4 µm for all the samples using an *in*-*situ* digital thickness monitor which was subsequently verified by a profilometer (Dektak 6, Veeco, USA). All films were post-annealed at 800 °C under the ambient condition to oxidize the film.

### Characterization

The crystallographic phase identification of the film was carried out using Bragg – Brentano (B-B) X-ray diffractometer operated at 40 keV using monochromatic Cu *Kα* radiation (λ = 1.5406 Å) with a scan rate of 2° per minute and at a step size of 0.02° (Rigaku Ultima IV, Japan). The pole figure analysis was carried out using high resolution x-ray diffraction technique (HR-XRD, D8 Advance, Bruker, Germany) by rotating the goniometer in both Ψ (0 to 90°) and ϕ (0 to 360°) direction. The recorded pole figure was processed using MTEX tool in Matlab software to quantify the orientation distribution function (ODF)^41^. Raman spectra were recorded in the range of 200 to 800 cm^−1^ at room temperature using confocal Raman spectrometer (Renishaw, UK) with a laser excitation wavelength of 514 nm and the acquisition time was maintained for 30 s. The surface morphology and cross-sectional view of the thin film were examined by a Field Emission-Scanning Electron Microscope (FE-SEM) with Carl Zeiss Ultra 55.

To analyze the electrical properties of the films, in-plane conductivity measurement were carried out with two-probe configuration using Novacontrol impedance analyzer (Alpha-A). Two parallel platinum electrodes were attached to the films by applying the platinum paste and conductivity was measured out in the air as a function of temperature from 400 to 850 °C at an interval of 50 °C. A soaking time of 20 minutes was provided at each temperature before beginning the impedance measurement. During the impedance measurement, a bias voltage of 1 V was constantly applied while sweeping the frequency from 1 Hz to 1 MHz.
